# Prevalence and intensity of *Ascaris lumbricoides* infections in relation to undernutrition among children in a tea plantation community, Sri Lanka: a cross-sectional study

**DOI:** 10.1186/s12887-018-0984-3

**Published:** 2018-01-25

**Authors:** Lahiru Sandaruwan Galgamuwa, Devika Iddawela, Samath D. Dharmaratne

**Affiliations:** 1 Department of Parasitology, Faculty of Medicine, University of Peradeniya, Peradeniya, Sri Lanka; 2 Department of Community Medicine, Faculty of Medicine, University of Peradeniya, Peradeniya, Sri Lanka; 3 Institute for Health Metrics and Evaluation, Department of Global Health, School of Public Health, University of Washington, Seattle, USA

**Keywords:** *Ascaris Lumbricoides*, Undernutrition, Children, Anthropometric measurements, Sri Lanka

## Abstract

**Background:**

*Ascaris lumbricoides* infections are one of the commonnest intestinal nematode infections in the world, with a profound negative effect on nutritional status among underprivileged populations. In Sri Lanka, *Ascaris* infections and low nutritional status still persist in the plantation sector. However, research regarding the association between *Ascaris* infections and nutritional status is scarce. The main purpose of this study was to determine the association between *Ascaris* infections and physical growth among children in a plantation sector in Sri Lanka.

**Methods:**

A cross sectional study was conducted among 489 children aged between 1 and 12 years ina plantation sector, Sri Lanka, from January to April 2013. Anthropometric measurements were collected to assess height-for-age (HAZ), weight-for-age (WAZ) and weight-for-height (WHZ) to determine stunting, underweight and wasting respectively. Data on socio-demographic and antihelminthic treatment were ascertained using an interviewer administrated structured questionnaire. Stool samples were subjected to wet mount preparation followed byformaldehyde-ether sedimentation technique to diagnose *Ascaris* infection and a Kato Katz technique was performed to determine the eggs intensity. AnthroPlus, EpiInfo and SPSS software was used to analyze data.

**Results:**

Of the study sample, 38.4% showed *Ascaris lumbricoides* infections. Light intensity infections (51%) were common in the infected children, followed by moderate (30%) and heavy (19%) infections. Prevalence of *Ascaris* infections was significantly associated with de-worming more than six months prior to the study. Prevalence of undernutrition among children was 61.7%. Forty-five per cent were underweight, while 24.1% and 21.5% of children were stunted and wasted respectively. However, no significant association was found between *Ascaris* infections status and undernutrition. Meanwhile, heavy intensity infections were associated with decreased values of WHZ (*p* = 0.020).

**Conclusions:**

*Ascaris* infections and undernutrition are still highly prevalent and a major public health problem in the plantation sector in Sri Lanka. Health and nutrition intervention programs should be implemented to increase the nutritional status of children.

## Background

*Ascaris lumbricoides* is a soil transmitted intestinal nematode common in low socio economic communities where limited access to clean water, poor personal hygiene and sanitary facilities is widespread [1]. It is estimated that more than 1 billion people are infected with intestinal parasitic infections worldwide and 10.5 million new cases are reported annually [2–4]. People of all ages are susceptible to infection, with the highest morbidity found in children due to low levels of acquired immunity and high exposure to contaminated soil [5]. *Ascaris* worms obstruct the small intestine in young children and occasionally enter and block pancreatic and bile ducts [6, 7], thereby causing malabsorption of vitamin A and reduction of lactose digestion. This leads to growth retardation, undernutrition, impaired cognitive functions and low educational achievements in children [8–10]. The occurrence of parasitic infections and undernutrition has a negative impact on growth and development of the infected person [11].

Nutrition is a major element of health and reflects the social and economic well-being of individuals and populations. Changes in body compositions affect the overall nutrition and health of a population. Undernutrition is one of the most common and persistent health problems in developing countries. It is an important underlying cause for more than half of child death [12, 13]. Undernutrition is often chronic, with deficits in dietary intake and high re-infection rates resulting in impaired physical and intellectual development [14, 15]. Anthropometry is widely used to assess health conditions and survival of individuals. It is a non-invasive and inexpensive measure of the nutritional status of an individual. Demographic and Health Survey (DHS) in 2006 reported that education level, household sanitation facilities and nutritional status were lower in the plantation sector, compared to urban and rural areas in Sri Lanka [16]. A recent study has reported that 35.6%, 26.9% and 32.9% of children in the plantation sector in Sri Lanka were categorized as underweight, stunted and wasted respectively [17]. In Sri Lanka, many studies reported that *Ascaris lumbricoides* was the commonest intestinal helminth infection among plantation sector school children [18–20]. However, information about the current health impact of *Ascaris lumbricoides* infection in Sri Lanka is insufficient. Hence, this study was designed to determine the prevalence and the intensity of *Ascaris* infection and the association with undernutrition in children in a tea plantation area of Sri Lanka.

## Methods

### Study area and population

A cross-sectional study was carried out in Uduwela tea plantation area (7^0^ 13′ − 7^0^ 17’ N and 80^0^ 37′ − 80^0^ 38′E) from January to April 2013. The study area is located in Kandy district and is 120 km away from Colombo, the capital city of Sri Lanka. It covers an area of about 2000 ha which is situated approximately 700 m above sea level, with an estimated population of 10,000 people in 2012. Annual average rainfall is between 2000 and 2500 mm and the average atmospheric temperature and humidity variations between 15 °C to 27 °C and 75% to 85% respectively. Most houses lack appropriate sanitation and portable water resources. As such, residents defecate in nearby jungles and retrieve water from streams and unprotected wells at low altitudes. Majority of residents in the study area are employed in tea plantations as unskilled laborers. This plantation area has been divided into seven sub-administrative divisions. Out of these divisions, three divisions were randomly selected by a lottery method. The target participants for the study were children in 1–12 years age group in the selected divisions. The sample size was calculated using the equation n = t^2^ × p (1-p)/ d^2^ where *n* = required sample size, *p* = estimated prevalence, *t* = 95% confidence level at 95% and d = margin of error at 5%. The estimated prevalence of *Ascaris* infections was taken as 30% in accordance with a recent survey of the plantation sector reporting a STH infection prevalence of 30% in children. On this basis, the minimal sample size was 323. As cluster sampling used in this study, a design effect was taken as 1.5.Thus the final sample size was 489.

### Collection of data

Before the commencement of the study, the objectives of the study were explained to all parents/guardians whose children were selected for the study. An interviewer administrated structured questionnaire was administered to collect socio - demographic characteristics (which included gender, age, number of rooms and family members) and data on de-worming practice. The questionnaire was designed in Sinhala and Tamil. Anthropometric measurements of height and weight were collected following Gibson’s guidelines using anthropometric rods and digital weighing scales to the nearest 0.1 cm and 0.1 kg, respectively in their homes [21]. Children stood on the floor without shoes and made sure that their shoulders, buttocks and the back of the head was in contact with the vertical backboard. Height of the selected children was measured as the maximum distance to the vertical position to the head from heels by using a stadiometer (SECA model 240). Weight was measured for children wearing light weight clothes, ensuring that their trunk was vertical above the waist and the arms and shoulders were relaxed.

A clean and dry wide mouthed container (with the identification number) was given to all the participants for the collection of stool samples. Parents and guardians were instructed to ensure that small proportions of stool were collected and the samples were not contaminated with water or urine. Stool samples were collected the following morning from their homes and transported to the Parasitology laboratory, University of Peradeniya within 2 hours of collection.

### Sample processing

Stool examination was conducted by experienced laboratory technicians at University of Peradeniya. Each stool sample was concentrated using formalin-ethyl acetate sedimentation technique [22]. Direct wet mounts were then prepared using Lugol’s iodine as well as in normal saline and examined microscopically under high power (× 40). Positivity of infection was based on the identification of *Ascaris* eggs. In addition, *Ascaris* eggs were quantified by Kato Katz technique to determine the intensity of infection [23]. Three slides made from each sample and every slide was read within 30–60 min. The recommended template of 41.7 mg was used and the average number of eggs counted was multiplied by 24 to get the number of eggs per gram of feces (EPG) [24]. Intensity of Ascaris infection was estimated as EPG (eggs per gram of feces) and classified into light, moderate and heavy with a definition of 1–4999 EPG, 5000–49,999 EPG and ≥50,000 EPG, respectively [25].

### Statistical analysis

Data was entered into Microsoft Excel 2007 sheet and cleaned to ensure accuracy (compared with questionnaires). Statistical analyses were performed using SPSS version 17 (SPSS, Chicago, IL, USA) statistical package. The descriptive data of continuous variables and proportions of categorical variables was expressed to obtain an understanding of socio-demographic characteristics of the study community.

Nutritional indicators were classified and standardized into sex-specific Z-scores for height-for-age (HAZ), weight-for-age (WAZ) and weight-for-height (WHZ) by WHO AnthroPlus 1.0.4. In addition, Z values for weight for height of children greater than 9 years old were determined using Epi Info 3.5.1. Children were classified as stunted, underweight and wasted when the Z scores of HAZ, WAZ and WHZ were less than - 2 standard deviation (< -2SD) respectively. This enabled estimation of the prevalence of undernutrition (i.e. underweight and/or wasted and/or stunted) in the study participants and subsequent evaluation of the effect of infection on this estimate. The association between the positivity of *Ascaris* infectivity with categorical variables (e.g., gender, age group, number of rooms, number of family members, deworming period, stunting, underweight and wasting) and continuous variables (e.g., HAZ, WAZ and WHZ) were calculated using the Chi square test (X^2^) and student t-test respectively. One-way ANOVA was applied to analyze differences in anthropometric mean Z-scores with the intensity of infections (light, moderate and heavy).

Multiple linear regression was used to determine the association between anthropometric indicators (HAZ, WAZ and WHZ) and infection status and intensity. Multiple logistic regression analysis was applied to determine the predictive effect of stunting, being underweight and wasting on the intensity and infection status. *Ascaris* infection status and the intensity were considered as dependent variables while socio demographic characteristics were considered as independent variables for both logistic and linear regressions. The differences were considered to be statistically significant when the *p* value was less than 0.05 at 95% confidence interval (CI) for the multivariate regression analysis.

### Ethical considerations

The ethical approval for the study was obtained from the Ethical Review Committee (ERC), Faculty of Allied Health Sciences, University of Peradeniya, Sri Lanka. Permission to conduct the study was given by the administrative authorities of the tea plantations and written informed consent was obtained from the parents/ guardian of the children who formed part of this study. Laboratory reports were issued with the signature of the Head of the department (Department of Parasitology) with recommendations. After the examination of stool samples, all children infected with ascariasis were administered anthelmintic treatments (single dose of 400 mg Albendazole) under the supervision of administrative and medical authorities.

## Results

### Prevalence and intensity of *Ascaris*infections

Four hundred eighty nine children with the mean age of 6.2 (SD = 3.4) years were recruited into the study and 249 (50.6%) of the participants were females. A total of five parasites were identified with *Ascaris lumbricoides* (38.4%, 188/489) followed by *Entamoeba coli* (16.6%), *Enterobius vermiularis* (2.0%), *Endolimax nana* (1.8%) and *Giardia lamblia* (0.2%). Children who lived in houses with one or two bedrooms and had more than five family members showed higher prevalence of *Ascaris* infection. Children who had taken anthelmintic treatments more than 6 months before conduction of the study were 1.6 times more likely to be infected than those were treated within last 6 months (*p* = 0.019). Approximately half of the infected children (51%, 96/188) were suffering from light intensity infections while 30% (57/188) and 19% (35/188) of children had moderate and heavy intensity infections respectively (Table 1).

**Table 1:** Risk factors associated with *Ascaris* infections (*n* = 489)

	Infection status			Infection intensity			
Variables	Positive	Negative	*p* value	Light	Moderate	Heavy	*p* value
Gender							
Male	91 (37.9)	149 (62.1)	0.903	48 (52.7)	27 (29.7)	16 (17.6)	0.977
Female	97 (39.0)	152 (61.0)		48 (49.5)	30 (30.9)	19 (19.6)	
Age groups (years)							
1–6	93 (38.8)	158 (61.2)	0.289	51 (54.8)	25 (26.9)	17 (18.3)	0.644
7–12	95 (39.9)	143 (60.1)		45 (47.4)	32 (33.7)	18 (18.9)	
No. of rooms							
1–2	81 (40.3)	120 (59.7)	0.482	46 (56.8)	19 (23.5)	16 (19.8)	0.289
> 2	107 (37.8)	181 (62.8)		50 (46.7)	38 (35.5)	19 (17.8)	
Family members							
≤ 5	75 (37.5)	125 (62.5)	0.721	42 (56.0)	18 (24.0)	15 (20.0)	0.480
> 5	113 (39.1)	176 (60.9)		54 (47.8)	39 (34.5)	20 (17.7)	
Deworming period							
≤ 6 months	67 (32.2)	141 (67.8)	0.015	31 (46.3)	23 (34.3)	13 (19.4)	0.074
> 6 months	121 (43.1)	160 (56.9)		65 (53.7)	34 (28.1)	22 (18.2)	

### Nutritional status of children

**Fig. 1:**
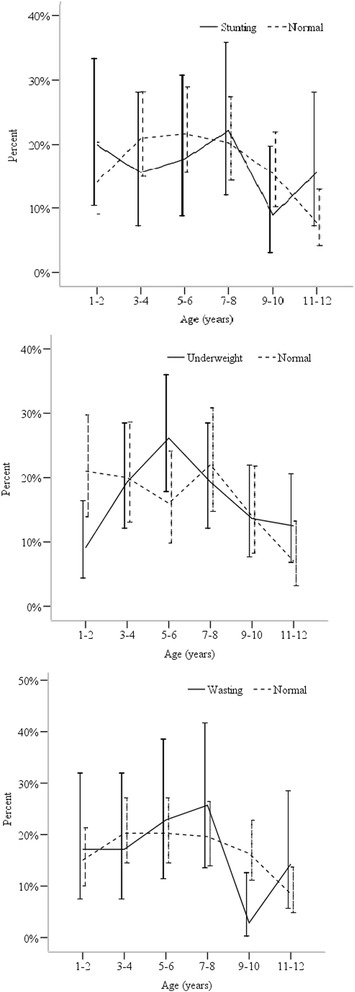
Prevalence of stunting, underweight and wasting among *Ascaris* infected children. (Vertical bars indicate 95% confidence interval)

**Fig. 2:**
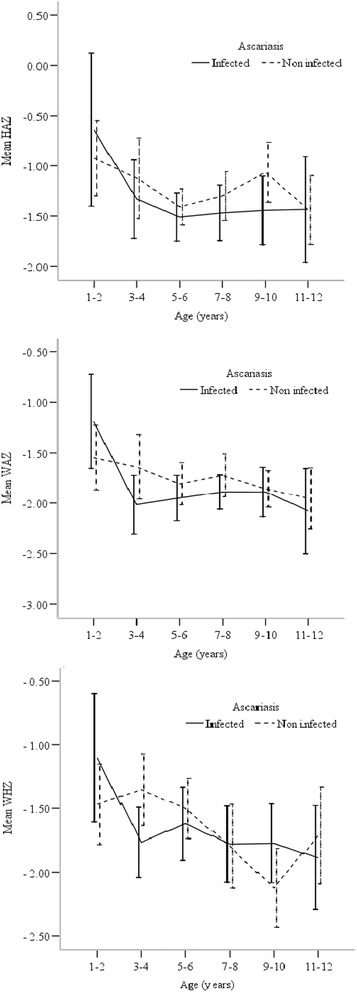
Mean Z scores of HAZ, WAZ and WHZ of infected and non-infected children. (Vertical bars indicate 95% confidence interval)

Prevalence of undernutrition among children in this community was 61.7% (302/489). The most common type of undernutrition in this community was being underweight (45%, 220/489) while 23.3% (*n* = 114) and 20% (*n* = 98) of children were stunted and wasted respectively (Fig. 1). However, no significant association was found between *Ascaris* infection status and undernutrition. Non-infected children had higher mean values of HAZ, WAZ and WHZ than infected children (Fig. 2). Results of the one-way ANOVA analysis revealed that mean values of nutritional indicators were not significantly associated with infection intensities of *Ascaris* infections (Table 2).

**Table 2:** Nutritional characteristics of the study group

	Infection status			Intensity of infections			
Nutritional indicators	Infected	Non-infected	*p*-value^b^	Light	Moderate	Heavy	*p*-value^c^
Stunted (< − 2 SD HAZ)	45 (39.5)	69 (60.5)	0.797	19 (42.2)	15 (33.3)	11 (24.4)	0.519
Underweight (<− 2 SD WAZ)	89 (40.5)	131 (59.5)	0.409	44 (49.4)	32 (36.0)	13 (14.6)	0.259
Wasted (< − 2 SD WHZ)	35 (35.7)	63 (64.3)	0.534	23 (65.7)	10 (28.6)	2 (5.7)	0.123
Mean HAZ score^a^	−1.32 (1.19)	− 1.21 (1.10)	0.309	− 1.13 (1.35)	− 1.39 (1.02)	− 1.69 (0.84)	0.055
Mean WAZ score^a^	− 1.83 (0.85)	− 1.74 (0.94)	0.276	− 1.82 (0.94)	− 1.89 (0.72)	−1.80 (0.82)	0.674
Mean WHZ score^a^	−1.65 (0.96)	−1.63 (1.08)	0.884	−1.28 (1.03)	− 1.62 (0.80)	−1.80 (0.92)	0.090

^a^For continuous variables, values in parentheses are the standard deviation

^b^Independent t-test used for continuous variables and chi-square test used for discrete variables

^c^One-way ANOVA used for continuous variables and chi-square test used for discrete variables

### Associations between STH infections and nutritional status

Multiple logistic regression revealed that age and gender was significantly associated with being underweight. Female children were 1.8 times more likely to be underweight (OR = 1.78; CI = 1.22–2.58, *p* = 0.003) than male children. Children in the age group of 7–12 year were 1.5 times (OR = 1.50; CI = 1.04–2.16, *p =* 0.030) more likely to be underweight. Children living with more than 5 family members were 1.6 times more likely to be stunted (OR = 1.61; CI = 1.02–2.53, *p =* 0.040) than those living with less than 4 family members. However, no demographic factors were found to be significantly associated with wasting in this study group. Infection intensity and/or infection status were not found to be significantly associated with nutritional indicators.

Estimated coefficients (β) from multiple linear regression models are shown in Table 3. Heavy infections with *Ascaris lumbricoides* were significantly correlated with a decrease in WHZ scores (β = − 0.20, 95% CI = − 0.31 to − 0.03, *p* = 0.020). However, the intensity was not correlated with HAZ and WAZ scores. Children of the female sex and those aged between 7 to 12 years showed significant negative correlations between WAZ (β = − 0.14, 95%CI = − 0.23 to − 0.05, *p* = 0.003and β = − 0.11, 95% CI = − 0.19 to − 0.02, *p* = 0.021 respectively). Conversely, HAZ and WHZ scores were not correlated with age and gender. Children who lived in houses with more than 2 rooms were positively correlated with WHZ and children with more than five family members were negatively correlated with HAZ (β = 0.08, 95% CI = 0.01 to − 0.15, *p* = 0.070 and β = − 0.09, 95% CI = − 0.16 to − 0.01, *p* = 0.046 respectively).

**Table 3:** Multiple analysis of nutritional status of the study group

		HAZ		WAZ		WHZ	
Variables	Category	Coefficient (β) (95% CI)	*p* value	Coefficient (β) (95% CI)	*p* value	Coefficient (β) (95% CI)	*p* value
Gender	Male	1		1		1	
	Female	− 0.05 (− 0.12, 0.04)	0.338	− 0.14 (− 0.23, − 0.05)	0.003	− 0.02 (− 0.09, 0.06)	0.668
Age (Years)	1–6	1		1		1	
	7–12	− 0.04 (− 0.11, 0.04)	0.413	− 0.11 (− 0.19, − 0.02)	0.021	− 0.02 (− 0.09, 0.02)	0.644
No. of rooms	1–2	1		1		1	
	> 2	0.05 (− 0.04, 0.12)	0.286	− 0.05 (− 0.14, 0.04)	0.305	0.08 (− 0.01, 0.15)	0.070
Family members	≤ 5	1		1		1	
	> 5	−0.09 (− 0.16, − 0.01)	0.046	0.01 (− 0.08, 0.10)	0.853	0.07 (− 0.02, 0.13)	0.143
Deworming period	≤ 6 months	1		1		1	
	> 6 months	0.03 (−0.05, 0.10)	0.507	0.03 (−0.06, 0.12)	0.524	−0.03 (− 0.10, 0.05)	0.501
Infection intensity	Negative	1		1		1	
	Light	−0.02 (−0.12, 0.08)	0.713	0.02 (−0.09, 0.14)	0.660	0.02 (−0.08, 0.11)	0.710
	Moderate	0.02 (−0.10, 0.15)	0.689	0.06 (−0.06, 0.23)	0.229	0.01 (−0.07, 0.09)	0.618
	Heavy	0.06 (−0.05, 0.24)	0.209	−0.04 (− 0.24, 0.10)	0.433	− 0.11 (− 0.31, − 0.03)	0.020
Infection status	Negative	1		1		1	
	Positive	0.01 (−0.07, 0.09)	0.820	0.03 (−0.06, 0.12)	0.510	−0.03 (− 0.10, 0.05)	0.455

## Discussion

According to the results of the present study, *Ascaris lumbricoides* infection is a major public health problem in the study group. This was relatively lower than previous research in plantation sector [19, 20, 24] and higher than studies conducted in rural areas and urban slums of Sri Lanka [26–28]. Several studies conducted in India [29], Malayasia [30] and Cuba [31]. have reported similar results. Poor sanitation and personal hygiene, poverty and lack of clean portable water in this study area could be probable reasons for the high prevalence of *Ascaris* infections noted in this study. Most children defecate indiscriminately in the backyards and bushes around dwelling places due to lack of latrine facilities. The eggs of *Ascaris* are more resilient helminth eggs that can remain in the infective stage for years embedded in the soil [32]. Therefore, soil pollution with *Ascaris* eggs is a major risk factor for the development of infection. As the eggs are very sticky, they readily adhere to raw fruits and vegetables, which are washed with contaminated water or fertilized with contaminated night soil. They may also circulate in household dust and air where they are inhaled or swallowed [33].

There was no significant difference in prevalence of *Ascaris* infection between males and females. This agrees with previous studies conducted in Sri Lanka [19, 24], as well as in Nigeria [34] and Brazil [35]. The prevalence and intensity of *A. lumbricoides* infection was higher in older children (7–12 years). Children of this age period often spend their leisure time outdoors, engaging in ctivities such as playing with sand and eating unclean food remains under the trees with unwashed hands. Therefore, the possibility of exposure to the infective stage of *Ascaris* parasites is higher among school children than pre-school children.

Unscheduled deworming practices are common in this community. Lack of anthelminthic drug availability and the absence of proper deworming programs in the plantation sector are major factors which contribute to high rate of re-infection in children of this community. Therefore, long-term interventions in the community level are necessary to reduce the prevalence and intensity of STH infections [36].

Values of nutritional indicators in a majority children observed in the present study were lower than the WHO standard values for growth and nutrition. However, similar results have been reported by the demographic and health survey (DHS) in 2006 which found that 42% of children under 5 years were stunted, 30% were underweight and 13% were wasted in the plantation sector of Sri Lanka [17]. Furthermore, a food and nutrition survey in 2012 reported that 13.1%, 19.6% and 23.5% of children under 5 years in Sri Lanka were stunted, wasted and underweight respectively. In addition, 8.9% and 28.3% of children aged between 5 and 10 years and 17.3% and 25.3% of children aged between 11 and 15 years were stunted and wasted respectively [37]. However, there was no significant association between *Ascaris* infections and the children’s nutritional status. Socio-economic factors and dietary habits could be major reasons for the high prevalence of parasitic infections and low nutritional status.

Low height-for-age index identifies stunting which is associated with long-term undernutrition, infections and poor food intake, subsequently affecting the cognitive development of the children [38]. Low level of education, especially among mothers, is a notable cause of poor nutritional status in the plantation sector. Poor nutrition from early childhood causes stunting in school going children [39]. In the present study, a large household size (more than 5 family members) was found to be at significantly higher risk of stunting possibly due to inadequate food supply for children in large households. Community based studies conducted in South Africa and Brazil reported that children living with more family members were more likely to be stunted [40, 41]. In addition, a study in rural Malaysia reported that stunting was associated with moderate-to-heavy intensity of *Ascaris* infections [42]. However, no significant association was found between stunting and intensity of infections among children in the studied population. Interestingly, we found that the risk of stunting among infected children decreased with age. This supports the reasoning that socio-economic factors and dietary habits are important factors influencing the nutrition status among children in this community.

Being underweight is an indicator of both short term (acute) and/or long term (chronic) undernutrition. In the present study, around half of the children (45%) were found to be underweight, which is similar to that reported in India [43, 44]. Low dietary intake, poverty and excessive energy outflows could be possible reasons for the higher prevalence of underweight children [45]. Mothers in the plantation sector work in tea estates during the daytime and have very limited time to feed their children. It could result in adequate intake of energy for children, especially at the age of 7–12 years, who need more energy for growth and development. We found that female children are at higher risk of being underweight than males.

Low weight-for-height index is a nutritional indicator of wasting for a specific age and is useful to determine children at risk and for assessing short-term (acute) nutritional changes. However, weight-for-height is usually not appropriate for evaluating changes in a population over longer time periods [46]. Failure to gain weight or weight loss due to poor dietary intake, disease, and infection can cause wasting [47]. High prevalence of wasting indicates a high prevalence of infections and acute undernutrition present in the study group. In the present study, any socio-demographic factors were not significantly associated with wasting.

With regard to Z scores, mean values of all nutritional indicators (HAZ, WAZ and WHZ) decreased rapidly from year two to year three and then relatively flattened until the age of 12 years. *Ascaris* infected children showed lower mean Z scores of anthropometric indices compared to non-infected children. In addition, we found that heavy intensity infections are significantly associated with negative values of WHZ. This is in contrast to a study in Honduras which reported that moderate-to-heavy infections were significantly associated with decreased values in WAZ and HAZ [48]. Low values of WHZ leads to loss of appetite and poor nutrient digestion and absorption, which finally results in decreased growth and development and poor cognitive function.

## Conclusions

*Ascaris lumbricoides* infection was found to be highly prevalent and is a major public health concern in this community. However, it is not a contributing factor for undernutrition. Integrated control programs based on chemotherapy in combination with health education and strong community involvement must be considered to decrease the prevalence of parasitic infections and the improvement of health and nutrition status. Future detailed research with long term follow up on the nutritional impact of *Ascaris* infection in this study area is required.

## Abbreviations

EPG: Eggs per grams
HAZ: Height-for- age
WAZ: Weight-for-age
WHZ: Weight-for-height

## References

[1] Elkins DB, Haswell-Elkins M, Anderson RM (1986). The epidemiology and control of intestinal helminthes in the Pulicat region of southern India. Study design and pre- and post-treatment observations on *Ascaris lumbricoides* infection. Trans Roy Soc Trop Med Hyg.

[2] Bethony JR, Brooker S, Albonico M, Geiger SM, Loukas A, Diemert D (2006). Soil-transmitted helminth infections: ascariasis, trichuriasis, and hookworm. Lancet.

[3] Crompton DW (2001). *Ascaris* and ascariasis. Adv Parasitol.

[4] De Silva NR, Brooker S, Hotez PJ, Montresor A, Engels D, Savioli L (2003). Soil-transmitted helminth infections: updating the global picture. Trends Parasitol.

[5] Galvani AP (2005). Age-dependent epidemiological patterns and strain diversity in helminth parasites. J Parasitol.

[6] Khuroo MS, Zargar SA, Mahajan R (1990). Hepatobiliary and pancreatic ascariasis in India. Lancet.

[7] Villamizar E, Mendez M, Bonilla E, Varon H (1996). De onatra S. *Ascaris lumbricoides* infestation as a cause of intestinal obstruction in children: experience with 87 cases. J Pediatr Surg.

[8] Taren DL, Nesheim MC, Crompton DW, Holland CV, Barbeau I, Rivera G (1987). Contributions of ascariasis to poor nutritional status in children from Chiriqui Province, Republic of Panama. Parasitology.

[9] Dickson R, Awasthi S, Williamson P, Demellweek C, Garner P (2000). Effects of treatment for intestinal helminth infection on growth and cognitive performance in children: systematic review of randomised trials. BMJ.

[10] Drake LJ, Jukes MCH, Sternberg RJ, Bundy DAP (2000). Geohelminth infections (Ascariasis, Trichuriasis, and hookworm): cognitive and developmental impacts. Semin Pediatr Infect Dis.

[11] Quihui-Cota L, Valencia ME, Crompton DWT, Phillips S, Hagan P, Diaz-Camacho SP, Tejas AT (2004). Prevalence and intensity of intestinal parasitic infections in relation to nutritional status in Mexican schoolchildren. Trans R Soc Trop Med Hyg.

[12] Schroeder DG, Brown KH (1994). Nutritional status as a predictor of child survival: summarizing the association and quantifying its global impact. Bull WHO.

[13] Pelletier DL, Frongillo EA, Schroeder DG, Habicht JP (1994). A methodology for estimating the contribution of malnutrition to child mortality in developing countries. J Nutr.

[14] Del Rosso JM. In school feeding programs: improving effectiveness and increasing the benefit to education: The Partnership for Child Development Publication. Oxford University press; 1999.

[15] Chopra M (2006). Mass de-worming in Ugandan children. BMJ.

[16] Department of Census and Statistics (2009). Sri Lanka demographic and health survey 2006/2007.

[17] Galgamuwa GLS, Iddawela WMDR, Dharmaratne SD (2017). Nutritional status and correlated socio-economic factors among preschool and school children in plantationcommunities, Sri Lanka. BMC Public Health.

[18] Sorenson E, Ismail M, Amarasinghe DKC, Hettiarachchi I, Dassanayake TS (1996). Prevalence and control of soil transmitted nematode infection among children and women in plantations in Sri Lanka. Ceylon Med J.

[19] Gunawardena K, Kumarendran B, Ebenezer R, Gunasingha MS, Pathmeswaran A, de Silva NK (2011). Soil-tranmittedhelminth infections among plantation sector school children in Sri Lanka prevalence after ten years of preventive chemotherapy. PLoS Negl Trop Dis.

[20] Galgamuwa GLS, Iddawela WMDR, Dharmaratne SD (2015). Development and piloting of Ascariasis surveillance system of children in Sri Lanka. Online J Public Health Informatics.

[21] Gibson RS (2005). Principle of nutrition assessment.

[22] Marti H, Escher E (1990). SAF–an alternative fixation solution for parasitological stool specimens. Schweiz Med Wochenschr.

[23] Katz N, Chaves A, Pellegrino J (1972). A simple device for quantitative stool thick-smear technique in schistosomiasismansoni. Rev Inst Med Trop São Paulo.

[24] Gunawardena GSA, Karunaweera ND, Ismail MM (2004). Socio- economic behavioral factor affecting the prevalence of *Ascaris* infection in a low-country tea plantation in Sri Lanka. Ann Trop Med Parasit.

[25] World Health Organization (2002). Prevention and control of intestinal parasitic infections: prevention and control of schistosomiasis and soil-transmitted helminthiasis. Technical report series no. 912.

[26] Fernando SD, Goonethilleke H, Weerasena KH, Kuruppuarachchi ND, Tilakaratne D, De Silva D (2001). Geo-helminth infections in a rural area of Sri Lanka. Southeast Asian J Trop Med Public Health.

[27] Karunaithas R, Murugananthan A, Kannathasan S (2012). Prevalence and associated factors of soil transmitted helminthes infection among preschool children of Vadamarachi educational zone. Ving Journal of science.

[28] De Siva NR, De Silva HJ, Jayapani VP (1993). Intestinal parasitoses in the Kandy area Sri Lanka. Southeast Asian J Trop Med Public Health.

[29] Traub RJ, Robertson ID, Irwin P, Mencke N, Andrew Thompson RC (2004). The prevalence, intensities and risk factors associated with geohelminth infection in tea-growing communities of Assam, India. Trop Med Int Health.

[30] Ngui R, Ishak S, Chuen CS, Mahmud R, Lim YAL (2011). Prevalence and risk factors of intestinal parasitism in rural and remote West Malaysia. PLoS Negl Trop Dis.

[31] Escobedo AA, Cañete R, Núñez FA (2008). Prevalence, risk factors and clinical features associated with intestinal parasitic infections in children from San Juan y Martínez, Pinar del Río, Cuba. West Indian Med J.

[32] Gilgen D, Mascie-Taylor CG. The effect of antihelmintic treatment of helminth infection and aneamia. Parastol 2000;122:105-22. 10.1017/s0031182000007113 11197758

[33] O’lorcain PHC, Holland CV (2000). The public health importance of *Ascaris lumbricoides*. Parasitology.

[34] Chukwuma MC, Ekejindu IM, Agbakoba NR, Ezeagwuna DA, Anaghalu IC, Nwosu DC (2009). The prevalence and risk factors of Geohelminth infections among primary school children in Ebenebe town, Anambra state, Nigeria. Middle-East J Sci Res.

[35] Nobre LN, Silva RV, Macedo MS, Teixeira RA, Lamounier JA, Franceschini SCC (2013). Risk factors for intestinal parasitic infections in preschoolers in a low socio-economic area, Diamantina, Brazil. Pathog Glob Health.

[36] Salam RA, Maredia H, Das JK, Lassi ZS (2014). Bhutta ZA community based interventions for the prevention and control of helmintic neglected tropical diseases. Infect Dise of Pov.

[37] Ministry of Health and UNICEF. National nutrition and micro nutrient survey: anaemia among children aged 6–59 months and nutritional status of children and adults. Ministry of Health. 2013. http://www.unicef.org/srilanka/MNS_Report. Accessed 28 Feb 2013.

[38] Shariff ZM, Bond JT, Johnson NE (2000). Nutrition and educational achievement of urban primary school children in Malaysia. Asia Pasic J Clin Nutr.

[39] World Food Programme (WFP) (2004). School children nutrition.

[40] Mamabolo RL, Alberts M, Steyn NP, Waal HA, Levitt NS (2005). Prevalence and determinants of stunting and overweight in 3-year-old black south African children residing in the central region of Limpopo Province, South Africa. Public Health Nutr.

[41] Cristina R, Israel P, Sa Leal V, Oliveira JS, Cristina S, Augusta LA (2011). Determinants of stunting in children under five in Pernambuco, Northeastern Brazil. Rev Saude Publica.

[42] Ahmed A, Mekhlafi HM, Al-Adhroey AH, Ithoi I, Abdulsalam AM, Surin J (2012). The nutritional impacts of soil-transmitted helminths infections among Orang Asli schoolchildren in rural Malaysia. Parasit Vectors.

[43] Medhi GK, Barua A, Mahanta J (2006). Growth and nutritional status of school-age children (6–14 years) of tea garden workers of Assam. J Hum Ecol.

[44] Osei A, Houser R, Bulusu S, Joshi T, Hamer D (2010). Nutritional status of primary schoolchildren in Garhwali Himalayan villages of India. Food Nutr Bull.

[45] Ramzan M, Ali I, Khan AS (2008). Body mass status of school children of Dera Ismail Khan, Pakistan. J Ayub Med Coll Abbottabad.

[46] Armstrong H, Lhotska L, Engle P (1997). The care initiative: assessment analysis and action toimprove care of nutrition.

[47] Stratton R, Green C, Elias M (2003). Disease-related malnutrition: an evidence-based approach to treatment.

[48] Ana Lourdes Sanchez AL, Jose Antonio Gabrie JG, Usuanlele MT, Rueda MM, Canales M, Gyorkos TW (2013). Soil-transmitted Helminth infections and nutritional status in school-agechildren from rural communities in Honduras. PLoS Negl Trop Dis.

